# MicroRNAs in Genitourinary Malignancies: An Exciting Frontier of Cancer Diagnostics and Therapeutics

**DOI:** 10.3390/ijms25179499

**Published:** 2024-08-31

**Authors:** Nikhita Kathuria-Prakash, Pranali Dave, Lizette Garcia, Paige Brown, Alexandra Drakaki

**Affiliations:** 1 Division of Hematology/Oncology, Department of Medicine, UCLA David Geffen School of Medicine, Los Angeles, CA 90095, USA; nkathuria@mednet.ucla.edu; 2 School of Medicine, California University of Science and Medicine, Colton, CA 92324, USA; pranalirdave@gmail.com; 3 Division of Hospice and Palliative Medicine, Department of Medicine, UCLA David Geffen School of Medicine, Los Angeles, CA 90095, USA; lizettegarcia@mednet.ucla.edu; 4 Department of Medicine, UCLA David Geffen School of Medicine, Los Angeles, CA 90095, USA; pebrown@mednet.ucla.edu

**Keywords:** MicroRNA, genitourinary cancers, prostate cancer, urothelial cancer, kidney cancer, testicular cancer, adrenocortical cancer

## Abstract

Genitourinary (GU) malignancies, including prostate, urothelial, kidney, testicular, penile, and adrenocortical cancers, comprise a significant burden of cancers worldwide. While many practice-changing advances have been made in the management of GU malignancies in the last decade, there is still significant room for improvement. MicroRNAs (miRNAs) are noncoding RNAs that regulate post-transcription gene expression and which have been implicated in multiple mechanisms of carcinogenesis. Therefore, they have the potential to revolutionize personalized cancer therapy, with several ongoing preclinical and clinical studies underway to investigate their efficacy. In this review, we describe the current landscape of miRNAs as diagnostics, therapeutics, and biomarkers of response for GU malignancies, reflecting a novel frontier in cancer treatment.

## 1. Introduction

MicroRNAs (miRNAs) are a family of small noncoding RNAs (ncRNAs) that regulate gene expression after transcription or at translation. MiRNAs range from 18 to 24 nucleotides (nt) in length, averaging 22 nt in humans [1]. It is estimated that 30% of human mRNAs are regulated by miRNAs at the 3′ untranslated region (UTR), and even more mRNAs are regulated by miRNAs when binding to the 5′UTR and the open reading frame (ORF) region are considered [2]. The outcome of interactions between miRNAs and their target mRNA molecules is dependent on bond complementarity and the expression levels of each molecule [3]. A single miRNA molecule can regulate many gene targets, and each mRNA molecule can be inhibited by different miRNAs [2]. Endogenous miRNAs support several cell processes, including proliferation, differentiation, apoptosis, metabolism, and DNA repair.

**Figure 1:**
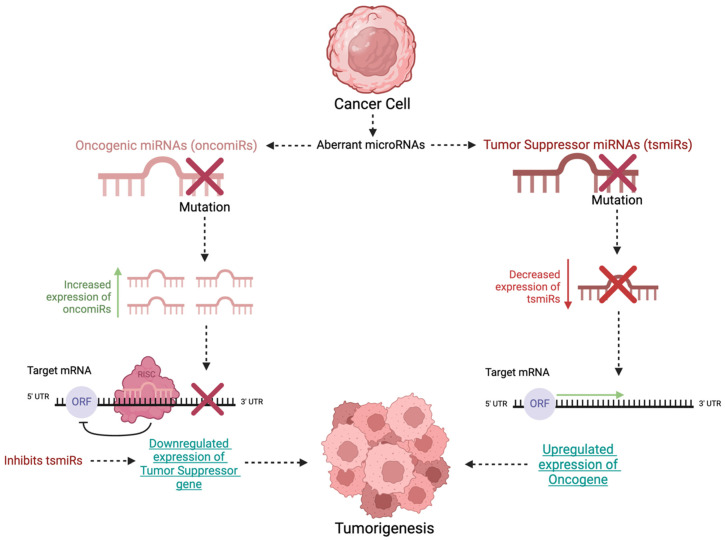
Aberrant microRNAs include oncogenic miRNAs (oncomiRs) and tumor suppressor miRNAs (tsmiRs) which can be mutated, resulting in downregulation of the tumor suppressor gene or upregulation of the oncogene, respectively.

Changes in miRNAs can induce carcinogenesis and cancer proliferation. Both increased and decreased expression of miRNAs have been implicated in many malignancies. MiRNAs can repress mRNA translation in humans through recognition of sequences in the 3′-UTR of mRNA that can be silenced by other ncRNAs [4]. Additional studies compared the miRNA of healthy and cancerous tissues and demonstrated that miRNAs can also induce the overexpression of target genes [5]. Therefore, tumor suppressor miRNAs inhibit the expression of oncogenes, whereas oncogenic miRNAs induce oncogenesis or inhibit the expression of tumor suppressor genes [6]. Two therapeutic strategies have been developed using miRNAs: synthetic miRNA replacement therapy and pharmacologic inhibition of oncogenic miRNAs. Replacement therapy is used to restore the expression of tumor suppressor miRNAs that are downregulated in cancer cells and involves treatment with synthetically produced suppressive miRNAs to inhibit oncogene expression. Conversely, the inhibition of oncogenic miRNAs prevents tumor growth (Figure 1) [7,8]. Disorders involving miRNA expression in cancer cells can be due to miRNA-encoding genes being located on sites that are fragile, sites that lack heterozygosity, or sites with minimal amplification regions [9]. Other causes include changes in transcriptional control, epigenetics, and miRNA biogenesis machinery.

Genitourinary (GU) malignancies include prostate, urothelial, kidney, testicular, penile, and adrenal cancers, comprising a significant burden of cancers worldwide [10]. The altered expression and function of miRNAs have been implicated in most GU malignancies [11]. Carcinogenic pathways targeted or facilitated by miRNAs in GU cancers include avoidance of apoptosis, promotion of cell proliferation, epithelial–mesenchymal transition (EMT), angiogenic signaling, and androgen independence [11,12]. While many practice-changing advances have been made in the management of GU malignancies in the last decade, significant work remains to be done.

MiRNAs are exciting biomarkers of and treatment options for GU cancers for several reasons. These include their availability in bodily fluids, their high biological stability, and their ability to regulate all stages of tumor development. Plasma or serum circulating miRNA values are useful early diagnostic tools for differentiating cancer patients from healthy individuals [12]. Furthermore, miRNA can help identify patients presenting with more aggressive cancers, promoting personalized treatment approaches. Such selective targeting could be the future of personalized cancer-directed treatment. In this review, we describe how novel miRNAs can be used in the treatment of prostate, urothelial, kidney, testicular, and other genitourinary cancers, both in preclinical and clinical settings, and describe areas of ongoing research for therapeutic indications of miRNAs in GU malignancies.

## 2. Prostate Cancers

Globally, prostate cancer remains the most common cancer in men [13]. It is the second most common cancer overall in the United States (US) and will represent an estimated 14.9% of all new cancer cases and 5.8% of all cancer deaths in 2024, according to projections made by the American Cancer Society [13]. Risk factors include advanced age, with over 75% of patients diagnosed over the age of 65, and race, with increased incidence in African American men. Usually, prostate cancer begins in the acinar cells of the prostate, known as prostate adenocarcinoma. Less common histologies include squamous cell carcinoma, mesenchymal tumors, and small cell prostate cancer [14]. Prostatic adenocarcinoma growth is typically driven by androgens. Therefore, the backbone of locally advanced or metastatic treatment includes androgen deprivation therapy (ADT) with gonadotropin-releasing hormone (GnRH) agonists or androgen blockade, while radical prostatectomy and radiation therapy with ADT are used in earlier stages of the disease [15]. In recent years, the use of theranostics, including the PSMA imaging modality, followed by treatment with Lutetium-177, have been employed as a new way of thinking outside of the box to create novel agents (other than chemotherapy and ADT) for the treatment of this disease [16].

While there has been significant progress in identifying circulating tumor cells and evaluating their role as a way to monitor disease activity, in the clinical setting, prostate specific antigen (PSA) serves as the main diagnostic tool, yet its limited specificity has led to many unnecessary invasive procedures [17]. MiRNAs have emerged as a promising area of research with potential to aid in both prostate cancer diagnosis and prognostication. Over 50 different miRNAs are involved in the development of prostate cancer through the dysregulation of both miRNAs involved in oncogenesis and tumor suppression [18,19]. In a genome-wide miRNA analysis by Haldrup et al. comparing serum samples from patients with benign prostatic hyperplasia (BPH) and patients with prostate cancer, miRNA-141 (miR-141) and miR-375 were overexpressed in patients with prostate cancer, and the degree of elevation may correlate with more advanced prostate cancer [20,21]. MiRNAs have also been studied as markers of prostate cancer treatment response. Cheng et al. correlated levels of miR-141, miR-200a, and miR-375 with circulating tumor cells (CTC) in patients enrolled in a phase II study. The study included 50 patients, exploring the effects of ADT+ cixutumumab versus ADT alone [22]. While all three miRNAs were associated with baseline CTC, a decrease in miR-375 was also associated with a decrease in PSA at 28 weeks, suggesting that miR-375 may be a useful biomarker for prostate cancer in the future [22]. Mechanistically, miR-375 controls repression of the tumor suppressor CBX7, which plays a role in epigenetic regulation [22]. Larger clinical trials are needed to further establish new markers of treatment response, especially in patients with metastatic castration-resistant prostate cancer (CRPC), in which the oncologist is often left to choose between few treatment options, without guidance regarding which patients may respond better to specific treatments. If certain miRNA signatures could predict which patients are more likely to respond to each treatment, clinicians could develop more personalized and effective treatment algorithms.

Distinct miRNA signatures have been found in patients with more aggressive prostate cancer, aiding in prognostication. Walter et al. conducted the profiling of tumor cells, comparing them to normal epithelium in 37 prostate cancer patients, and found the upregulation of miR-143 and miR-146b and the loss of several miRNAs in the tumor cells [23]. Later studies have found that miR-143 targets AKT to inhibit EMT, and thereby, upregulation leads to oncogenesis (doi 10.3390/cells12182207). MiR-146b also targets the PTEN/AKT/mTOR signaling pathway, thus inhibiting autophagy and promoting tumor growth (doi 10.18632/aging.101534). Predicting which patients may present with more aggressive malignancies can also aid clinicians in refining treatment algorithms, e.g., helping them determine when to consider triplet therapy with combination ADT and chemotherapy, and guiding discussions with patients regarding prognosis.

Given that prostate adenocarcinoma is largely driven by androgens, it is not a surprise that several miRNAs modulate androgen receptor (AR) signaling. Mishra et al. demonstrated that the interplay between miR-21 and AR reduces the growth-inhibitory effects of transforming growth factor β (TGFβ), in effect leading to prostate cancer progression [24]. Similarly, androgen regulation by miR-32 has been shown to reduce apoptosis. This was seen in in vitro studies using the lymph node carcinoma of the prostate (LNCaP) cell line that showed reduced expression of tumor suppressor B-cell translocation gene 2 (BTG2) in cells transfected with pre-miR-32 [25]. In addition, androgen-induced miR-135 regulates prostate cancer apoptosis through suppression of the RB-associated KRAB zinc finger (RBAK) and limits prostate cancer migration through the downregulation of matrix metalloproteinase 11 (MMP11) [26]. This provides insight into a potential mechanism of tumor resistance in CRPC, where androgen deprivation may paradoxically result in loss of tumor suppression. MiRNAs regulate castration resistance through different mechanisms, and these have become a target in the development of therapeutics for this disease, which currently has limited treatment options. These mechanisms include apoptosis avoidance, interference in androgen signaling, EMT, cancer stem cells, and multidrug resistance transporters [27].

MiRNA therapeutic strategies for prostate cancer have been studied in vivo and in vitro. The common goal is to reduce the expression of oncogenic miRNAs and restore tumor suppressor miRNAs. One method involves silencing miRNA using anti-miRNA oligonucleotides (AMOs) [28]. AMOs bind to complementary sequences of their target mature miRNA and block the miRNA-guided action of RNA-induced silencing complex (RISC) on tumor suppressor mRNA [28]. To function in vivo and in vitro, AMOs are chemically modified, which is usually completed in the C2′ position [28]. Krutzfeldt et al. developed AMOs to target prostate cancer, known as antagomirs [29]. Mercatelli et al. showed that in vitro inhibition of miR-221 and miR-222 limits tumor growth in the prostate cell line PC3 [30]. Next, they injected miR-221 and miR-222 antagomirs into PC3-derived tumors in mice, which led to the upregulation of p27 and decreased tumor growth [30]. This suggests that miR-221 and miR-222 upregulate p27, which may be an important tumor suppressor in prostate cancer.

MiRNA replacement therapy involves the delivery of chemically modified miRNAs mimics to cells, where they are dysregulated, in effect restoring the function of endogenous tumor suppressor miRNAs. These are usually chemically modified to increase their effectiveness. A common modification includes 2′O-methylation [31]. Gaur et al. demonstrated that the delivery of miR-34 into a prostate tumor mouse model led to the downregulation of MET, Axl, and c-Myc, resulting in decreased tumor growth and apoptosis in miR-34 treated cells compared to that noted in the controls [32]. Further, injection of miR-34 reduced prostate cancer bone metastasis growth in an intra-femur tumor mouse model [32]. This shows that miR-34 plays an important role in prostate cancer bone metastases and may be a clinical target for patients with bone metastases. Similarly, Hao et al. used an aptamer (APT)-conjugated atelocollagen (ATE) to deliver miR-15 and miR-16 in a mouse model of human prostate bone metastasis, which led to improved survival times and demonstrated an anti-tumor effect [33]. Moreover, the miRNA/ATE-APT complex proved to be more efficacious and to exert a greater anti-tumor effect than the miRNA/ATE complex, thereby demonstrating that this delivery mechanism may be more biocompatible and may exhibit greater potential for clinical translation [33].

The delivery mechanism has proven to be an important limitation of these miRNA therapeutic strategies, e.g., unmodified miRNAs are susceptible to quick degradation by nucleases and are rapidly cleared. This is one of several challenges in the clinical translation of miRNA-based therapies. Thus, improved carriers are needed to better target the tissue of interest and achieve a therapeutic effect [34]. Another challenge is that the introduction of miRNAs activates the innate immune system, leading to rejection of the introduced material. Additional challenges include creating miRNAs that are selective to their targets, as well as mitigating toxicity and off-target effects (doi 10.3390/ijms25031469). There are also economic challenges involved in scaling miRNAs to be viable options for cancer treatment. MiRNAs remain a promising area in prostate cancer research, particularly in regards to castration resistance, which currently presents limited treatment options. However, despite the progress in miRNA research, the inconsistency of specific miRNA patterns and the limited reproducibility of the studies have constrained their clinical translation, and therefore, miRNA therapeutics have yet to be tested clinically in prostate cancer.

## 3. Urothelial Cancers

Bladder cancer is one of the top ten most common cancers globally, with 573,000 new cases and 213,000 deaths in 2020 [35]. The vast majority of all bladder cancers consist of urothelial carcinoma, also known as transitional cell carcinoma; however, other rare histologies like squamous cell carcinoma, adenocarcinoma, and small cell carcinoma could co-exist. Urothelial carcinoma can originate anywhere in the urothelial tract, including from the lining of the bladder, urethra, ureters, and renal pelvis. While many advances have recently been made in the treatment of urothelial cancers, significant work remains to be done.

MicroRNAs (miRNAs) have primarily been studied as blood or urine biomarkers for urothelial cancer, both as diagnostic and prognostic tools [11,36,37,38,39,40,41]. Similar to the results for prostate cancer, studies have found that miRNAs can help identify patients presenting with more aggressive bladder cancers, and thus, they may be able to predict those patients which would benefit from adjuvant treatment or those that may demonstrate a sufficient response to a given treatment [36,37,38]. MiR-145 is of specific interest in bladder cancer as a promising biomarker; the methylation of the miR-145 core promoter results in the methylation of ZNF154 [42]. Pretreatment cell-free DNA methylation of the miR-145 core promoter was associated with increased risk for short-term progression and poorer survival of patients with muscle invasive bladder cancer (MIBC) after surgery and adjuvant therapy [42]. These advances suggest that miRNA is a promising diagnostic and prognostic tool for urothelial cancer, spurring interest in miRNA as a therapeutic target.

Various preclinical studies have identified specific miRNAs involved in carcinogenesis and have demonstrated efficacy in in vitro and in vivo models reintroducing synthetic versions of the miRNA. Xu et al. identified miR-100 as a direct inhibitor of human bladder urothelial carcinogenesis by targeting mTOR [43]. They found miR-100 to be downregulated in bladder cancer tissues, and when the expression of miR-100 in bladder cancer cells was ectopically restored, cell proliferation was suppressed, and tumorigenesis was inhibited in vivo [43]. These studies suggest that miR-100 may be a tumor suppressor in bladder cancer, and the reintroduction of miR-100 into tumor tissue may be a therapeutic strategy [43]. Similarly, Uchino et al. studied miR-582-5p and -3p and found that both are downregulated in MIBC, and transurethral injections of synthetic miR-582 suppressed tumor growth and metastasis in their animal model [44]. The authors also identified the target genes of miR-582, namely protein geranylgeranyltransferase type I beta subunit (PGGT1B), leucine-rich repeat kinase 2 (LRRK2), and DIX domain containing 1 (DIXDC1), and suggest that these two strands of miRNA are a potential therapeutic target to treat MIBC [44]. MiR-122 represses VEGFC post-transcriptional expression which decreases AKT and mTOR, and miR-122 was identified by Wang et al. as downregulated in bladder cancer [45]. They found that miR-122 prevents angiogenesis, and when cells overexpressing miR-122 were injected into bladder cancer mouse models, angiogenesis and cancer growth were slowed [45]. They also found that cells injected with miR-122 were very sensitive to cisplatin-induced apoptosis, suggesting that miR-122 could be used as an adjunct in combination with cytotoxic chemotherapy to increase its efficacy [45]. Lastly, Wang et al. identified miR-124 to be downregulated in bladder cancer tissues, and the overexpression of miR-124 decreased tumor growth in xenograft models by suppressing the expression of UHRF1 through binding its 3′-UTR, an epigenetic factor [46].

**Figure 2:**
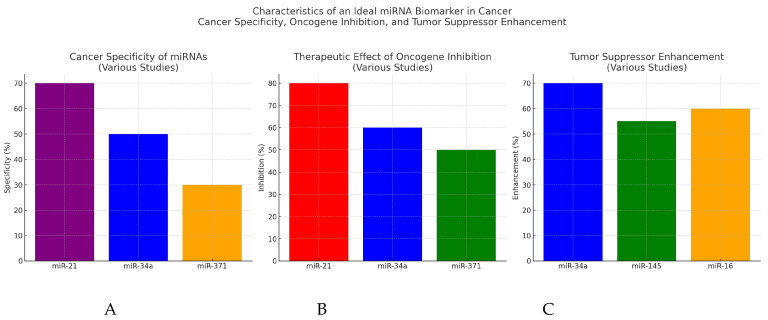
Graphical depiction of the characteristics of an ideal miRNA biomarker in cancer. (**A**) Specificity of cancer detection for miR-21, miR-34a, and miR-371, respectively. (**B**) Percentage of inhibition of oncogenes by miR-21, miR-34a, and miR-371, respectively, demonstrating the therapeutic effect of each oncogene. (**C**) Percentage of enhancement of the tumor suppressor effect of miR-34a, miR-145, and miR-16, respectively.

A few studies have identified miRNAs involved in urothelial cancer development and trialed investigational therapies in vitro and in vivo, demonstrating success in inhibiting tumor growth. Drakaki et al. first found that miR-21 was upregulated fivefold in bladder cancer tissues compared to normal tissues, and that expression was increased in advanced relative to early stage bladder tumors, suggesting potential involvement in bladder cancer progression (Figure 2) [47]. Next, they demonstrated that treatment with locked nucleic acid miR-21 (LNA miR-21) reduced the ability of bladder cancer cells to form colonies in vitro [47]. Subsequently, they treated a bladder cancer xenograph mouse model with LNA miR-21 and were able to replicate their findings in vivo [47]. Interestingly, they found that increased levels of miR-21 expression correlated with treatment response, suggesting miR-21 may be a therapeutic target and a potential biomarker to monitor response [48,49]. Next, they developed an antisense oligonucleotide against miR-21, called ADM-21, and evaluated its efficacy in vitro and in vivo [48]. They demonstrated that intravenous administration of ADM-21 into mouse xenografts reduced the tumor growth rate by 37–47% after three cycles [48,49]. The authors identify that the target of miR-21 is PPP2R2A, a negative regulator of the ERK pathway which promotes bladder cancer growth [49]. The authors concluded that ADM-21 may be an effective treatment for bladder cancer, pending further evaluation in clinical trials [48]. Heishima et al. similarly studied miR-145 and found that miR-145 regulates interferon pathways and c-Myc expression, and that the expression of miR-145 is downregulated in premalignant lesions of non-muscle invasive bladder cancer (NMIBC) (Figure 2) [50]. They created a novel miR-145-based intravesical agent and demonstrated that treatment with synthetic miR-145 therapy inhibited the growth and disease progression of premalignant lesions in mouse models, without significant systemic leak or adverse effects [50].

**Figure 3:**
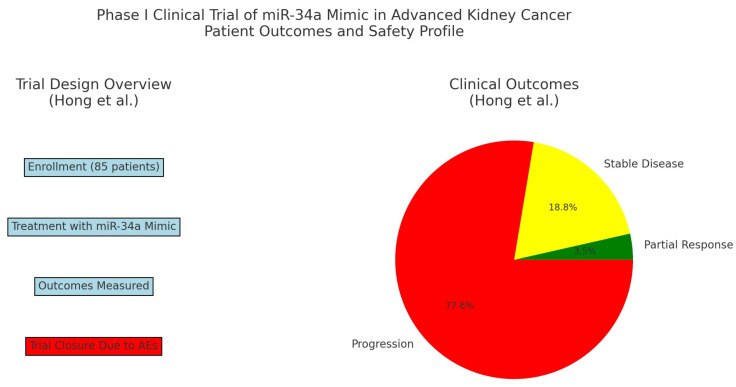
Graphic depiction of the trial design and clinical outcomes from the investigation of miR-34 as a therapeutic agent (from Hong, et al.) [51].

Lastly, Hong et al. reported the results of a first-in-human phase 1 study of a micro-RNA based cancer therapy, MRX34, in patients with advanced solid tumors, including patients with bladder cancer [51]. They enrolled 85 patients with various malignancies and administered a liposomal mimic of miR-34a, a tumor suppressor involved in the pathogenesis of several tumor types (Figure 2) [51]. A total of 3 patients exhibited partial responses, and 16 patients showed stable disease lasting at least four cycles, with a median duration of response of 19 weeks (Figure 3) [51]. The toxicity profile was manageable, but the trial closed early due to serious immune-mediated adverse effects, resulting in the death of four patients [51]. However, the authors concluded that this study provides a proof-of-concept for miRNA-based cancer therapy [51]. Although the authors did not report the outcome of the patient with bladder cancer specifically, this study provides the only clinical data thus far for miRNA as a therapeutic modality or target for bladder cancer.

Currently, the trial by Hong et al. provides the only completed clinical investigation of miRNA-based cancer therapeutics [51]. As most miRNA-based therapies are still in the preclinical stages, it will likely be several years before miRNA-based regimens are used as standard of care cancer treatments. It is important to note the significant differences between miRNA-based treatments and conventional cancer treatments, such as chemotherapy and radiation therapy. Chemotherapy agents have nonspecific mechanisms that kill rapidly dividing cells, such as inhibiting microtubules or topoisomerases, causing DNA damage by introducing alkylated nucleotides and preventing replication, or inducing double stranded DNA breaks at active promotor regions. Ionizing radiation also results in DNA damage. Therefore, both chemotherapy and radiation therapy result in the death of the cancer cells, but also in significant side effects due to the damage of healthy cells that are actively dividing at the time of administration. On the contrary, miRNA-based cancer therapies are expected to be more specific in targeting cancer cells, as the miRNA target is an mRNA or protein that is specifically involved in oncogenesis. Synthetic miRNA replacement therapies could replace the tumor suppressor miRNAs that are mutated in cancer cells, and oncogenic miRNAs could replace mutated oncogene miRNAs. These treatments would be much more specific solely to the cancer cells that are missing these miRNAs and therefore, would not exert the off-target effects that can cause severe adverse events from chemotherapy and radiotherapy. Rather, we anticipate that the adverse events with miRNA-based therapies would be immune-related adverse events, similar to the adverse effects from cellular therapies or bispecific antibodies, given that these were the serious adverse events reported by Hong et al. [51]. However, these adverse effects would have to be diminished or mitigated to advance clinical investigation of miRNA-based therapy, since immune-related adverse events were the limiting toxicity of this phase I study [51]. Given the identification of several other miRNAs involved in the oncogenesis of urothelial cancer since this clinical trial, we anticipate that future phase 1 studies will continue to investigate this exciting new treatment modality in urothelial cancers, which affect hundreds of thousands of people yearly worldwide.

## 4. Kidney Cancers

Kidney cancer, also known as renal cell carcinoma (RCC), is the 7th most common neoplasm in the developed world, with 403,000 cases globally per year [52]. The most common RCC histology is clear cell, followed by papillary, chromophobe, and unclassified types. RCC is not a chemosensitive malignancy; therefore, treatment is typically multimodal, employing surgical resection and adjuvant immune checkpoint inhibitors (ICI) for high-risk completely resected localized disease [53]. Single agents, or a combination of ICIs with vascular endothelial growth factor tyrosine kinase inhibitors (VEGF TKIs), are the mainstay of metastatic disease, while more recently, a hypoxia inducible factor (HIF) inhibitor has been added to the effective therapies, along with the mammalian target of rapamycin (mTOR) inhibitors in the third-line setting [53]. Despite the scientific progress and the FDA approval of dozens of new treatment drugs over the past decade, metastatic kidney cancer remains a fatal disease, and novel agents with different mechanisms of action are needed [54]. MiRNAs are involved in multiple signaling pathways associated with oncogenesis in RCC, and both the downregulation and upregulation of these can promote cancer formation and progression, while some of them could be used as diagnostic tools [55]. MiR-21 is upregulated in RCC, targeting PTEN and PDCD4, and miR-106a is downregulated, targeting PAK5 in the RAS/MAPK signaling pathway; the serum levels of both are higher in patients with RCC compared to those of the controls, and levels decreased after RCC resection [55,56]. Additionally, miR-342, miR-130a, and miR-30c-2 may exhibit prognostic utility in RCC, as overexpression of these has been associated with poorer survival [55,57].

Several in vitro studies have demonstrated that miRNAs can mediate treatment resistance to chemotherapy and VEGF inhibitors in RCC. MiR-144-3p targets ARIDIA, miR-141 targets ZEB2, and miR-130b and miR-96-5p target PTEN; all promote sunitinib resistance in RCC by over- or under-expression, thus regulating tumor suppressors and oncogenes to promote tumor proliferation [55,58,59,60,61]. MiR-31-5p targets MLH1, miR-200C targets HO1, and miR-195-5p targets the wnt/Beta-catenin pathway; all promote sorafenib resistance through increasing tumor proliferation and decreasing apoptosis [62,63,64,65,66,67,68]. Similarly, miR-30c, miR-451, miR-489-3p, miR-630, and miR-21 increase resistance to various chemotherapeutics [62,63,64,65,66,67,68]. While chemotherapy is no longer recommended to treat RCC, and sunitinib and sorafenib have since been replaced by newer generation VEGF TKIs as first-line treatment options, miRNAs may modulate treatment efficacy in newer VEGF TKIs as well, and may explain why certain patients’ tumors are more sensitive to treatment compared to others [54]. Potential therapeutic mechanisms include the introduction of a synthetic mimic of miRNAs that are downregulated in VEGF TKI resistance or the development of an inhibitor of miRNAs that promote VEGF TKI resistance. MiR-141 induces hypoxia resistance and EMT, but Berkers et al. noted the reversal of the hypoxic conditions promoted by the downregulation of miR-14 when miR-141 was reintroduced in vitro, suggesting that sunitinib resistance could be reversed [59]. Similarly, Yang et al. demonstrated that when miR-30 was injected into the RCC model, MTA-1 was repressed, and the sensitivity of the cells to sorafenib and paclitaxel was significantly enhanced [65].

MiRNAs have also been implicated in predicting sensitivity to ICI. Chen et al. demonstrated that treatment with miR-381 increased the sensitivity of RCC cells to 5-fluorouracil (5-FU) in vitro; although 5-FU is not recommended for treatment of RCC, this data may be useful if replicated in other cancer types in which 5-FU remains the standard of care, and they suggest that miRNA plays a pivotal role in modulating the cytotoxic effects of chemotherapy [69]. ICI has revolutionized the treatment of RCC, and Ivanova et al. described that the exosomal miRNA expression profiles of patients with RCC significantly differed before and after treatment with ICI, suggesting that this miRNA signature can be used as a predictor of ICI therapy effectiveness [54,70]. The authors evaluated the expression of miR-144, miR-146a, miR-149, miR-126, and miR-155 in 35 patients with RCC treated with ICI and found that miR-146a levels were significantly increased after treatment, but were lower in patients with a higher grade of immune-related adverse effects [70]. MiR-126 levels were significantly reduced after therapy, and the sensitivity and specificity of the combination of miR-146a and miR-126 for predicting ICI response were 64.3% and 78.9%, respectively, providing preliminary data that miRNAs can be powerful predictors of the effectiveness of ICI [70]. The first-line treatment of metastatic RCC is currently VEGF TKI/ICI combination therapy or dual ICI therapy; a prognostic biomarker for determining which patients may benefit from up-front doublet ICI therapy versus TKI could transform RCC treatment and promote more personalized cancer care [53].

MiRNAs also regulate RCC growth and activity by modulating signaling pathways involved in cancer progression; the reintroduction or inhibition of various miRNAs has been tested in vivo and in vitro. Zhao et al. demonstrated that miR-187 targets B7-H3 to inhibit cell growth and migration, and miR-187 was downregulated in the tumor tissue and plasma of clear cell RCC patients [71]. Lower miR-187 expression levels were associated with higher tumor grade and stage [71]. Additionally, when RCC cells were treated with the miR-187 expression vector, in vitro proliferation was suppressed, and in vivo tumor growth was inhibited, suggesting that synthetic miR-187 could be administered as a tumor suppressor, or that miR-187-based gene therapy may be developed as a treatment of RCC [71]. In a similar experiment, Kalantzakos et al. found that the downregulation of miR-155-5p increased levels of the tumor suppressor Jade-1 and decreased the rate of tumor implantation and proliferation in a mouse xenograft model, and that the knockout of miR-155-5p decreased the rate of tumor implantation [72]. Liang et al. found that miRNA-21 promoted proliferation and differentiation and decreased the apoptosis of RCC cells by targeting the mTOR pathway [73]. Next, they treated human RCC cell lines with either a miR-21 mimic, an mTOR inhibitor, or both, and evaluated the activity of caspase 3, an apoptotic protein. The mTOR inhibitor increased caspase 3 activity, whereas the miRNA-21 mimic inhibited caspase 3 activity, suggesting that an mTOR inhibitor may downregulate and inhibit the oncogenic effects of miR-21 [73]. Everolimus and temsirolimus are mTOR inhibitors approved to treat metastatic RCC, and their therapeutic effects may include the downregulation of miR-21 [53].

Another potential therapeutic target mediated by miRNAs includes epigenetic modification and the tumor microenvironment of RCC. MiRNAs regulate the tumor microenvironment in RCC by mediating the interactions between the cancer cells, the extracellular matrix, the signaling molecules, and various immune and non-immune cells [74]. As research continues, miRNAs in the RCC tumor microenvironment offer another potential therapeutic target. Additionally, miRNAs are involved in epigenetic modifications that promote carcinogenesis, and Schiffgen et al. demonstrated that treatment of RCC cell lines with inhibitors of DNA-methyltransferase and histone-deacetylase promoted the effects of multiple miRNAs [75]. Specifically, histone acetylation at the miR-9-1 promoter region was considerably increased when treated with a combination of epigenetic regulators, resulting in tumor suppression mediated by miR-9-1 [75]. In a similar study, Wu et al. reported the downregulation of miR-492 in RCC tissues due to hypermethylation of the miR-492 promoter [76]. When treated with DNA demethylating agents or histone deacetylase inhibitors, miR-492 was significantly upregulated in RCC cells, inhibiting cell proliferation and invasion [76]. The authors conclude that miR-492 may be a promising therapeutic target, specifically through epigenetic regulation [76]. MicroRNAs are involved in RCC disease progression and present a powerful therapeutic target. While data is thus far limited to the preclinical setting, evidence is building to suggest that clinical studies should be conducted to evaluate miRNAs as a new treatment option for RCC.

## 5. Testicular Cancers

Testicular cancer is the most common malignancy among men aged 15 to 45 years old [77]. When diagnosed early, testicular cancer is curable in over 90% of cases, with more than 95% of patients surviving beyond 5 years [78]. Testicular cancers can be broadly divided into germ cell tumors (GCTs) and non-germ cell tumors (NGCTs). GCTs make up approximately 95% of testicular cancers and can be further subdivided into seminomas and nonseminomatous tumors, including embryonal carcinoma, choriocarcinoma, yolk sac tumors, and teratomas. In 2022, there were approximately 72,000 new cases and 9,000 deaths due to testicular cancer worldwide [79]. Although testicular cancer is considered one of the most curable malignancies, the incidence has been increasing globally, emphasizing the importance of ongoing research, as patients with refractory disease experience short survival.

Various pre-clinical studies have demonstrated promising results for the use of miRNAs in the early diagnosis and therefore, the management, of GCTs. While the serum tumor markers alpha fetoprotein (AFP), beta-human chorionic gonadotropin (β-hCG), and lactate dehydrogenase (LDH) are widely utilized in testicular cancer, miRNAs are under investigation as promising markers for diagnosis, prognosis, therapeutic monitoring, and surveillance. Due to their contribution to oncogenesis through mechanisms such as angiogenesis, metabolism, immune activation, and tumor invasion, miRNAs are thought to serve as useful biomarkers that may be more sensitive and specific than AFP, β-hCG, and LDH [80]. Biomarkers that have been of particular interest in testicular cancers include, but are not limited to, miR-371-3p (miR-371), miR-302, miR-367, and miR-320.

MiR-371 is the most studied of the miRNAs associated with TGCTs, although its target in TGCTs is not well-described. MiR-371 is elevated in both seminomas and nonseminomatous GCTs but is not expressed in teratomas [81]. Peripherally circulating miR-371 was first documented by Murray et al. in both adult and pediatric patients [82]. Their studies revealed that miR-371 was elevated in patients with GCTs and not in healthy controls, and variation in the level of miR-371 across GCT subtypes was observed (Figure 2) [82]. They also demonstrated the feasibility of measuring serial serum miR-371 levels, as well as close correlation of those levels with treatment response [81,82]. Several studies have also demonstrated a positive correlation between the size of GCT primary lesions and the level of miR-371; the sensitivity was the greatest for nonseminomatous lesions compared to that for pure seminomas [82,83,84,85]. Belge et al. conducted a study of 258 patients with clinical stage I GCT, assessing miR-371 as a marker for detecting relapse [86]. All patients with disease relapse exhibited elevations of miR-371, and the study described the following characteristics of miR-371 to predict GCT relapse: area under the receiver operating characteristics curve of 0.993, sensitivity of 100%, specificity of 96.3%, positive predictive value of 83%, and negative predictive value of 100% [86]. In a comprehensive review, Nestler et al. summarized these findings and suggested five key applications of miR-371: (1) enhanced diagnostic workup with the potential of sparing surgical exposure in selected small neoplasms, (2) early detection of relapses, (3) enhanced diagnostic workup of retroperitoneal lymphadenopathies, potentially avoiding diagnostic surgery or advanced imaging techniques, (4) identification of chemotherapy non-responders, and (5) assistance with management decisions in post-chemotherapy residual disease [81].

Unlike miR-371, the miR-302 cluster is mainly found in teratomas [87]. MiR-302 suppresses AKT1 expression, promoting the pluripotent factor OCT4 [87]. This miRNA also interacts with cell cycle promoters and inhibitors and regulates processes such as histone methylation and other signaling cascades [87,88]. Das et al. demonstrated an increase in serum miR-302 in patients with GCT in contrast to low expression in patients with colon, stomach, and liver carcinomas [89]. MiR-302 levels also decreased when exposed to cisplatin [89]. The miR-367 cluster differs slightly in sequence from the miR-302 cluster but has similar mRNA targets [89]. A study by Syring et al. demonstrated that miR-367 was significantly increased in patients with GCT compared to healthy patients, was lower in early-stage tumors compared to advanced tumors, was higher in seminomas compared to nonseminomatous tumors, and decreased or became undetectable after orchiectomy [90]. MiR-320 is a novel miRNA first reported in 2017 as a suppressor of gliomas, and it has since been implicated in various cell signaling pathways that impact testicular cancer development [91,92,93].

As is the case in RCC, miRNAs also play a role in enhancing or inhibiting chemotherapy resistance in GCTs. For example, miR-302 induces apoptosis and enhances sensitivity to cisplatin through p21 downregulation [94]. MiR-383, miR-106b-5p, and miR-514a-3p also inhibit cisplatin resistance [94]. Conversely, miR-512-3p, miR-515, miR-517, miR-518, miR-525, miR-99a, miR-100, and miR-145 promote cisplatin resistance [94].

There are currently no completed clinical trials investigating miRNAs in testicular cancer, but several are ongoing, primarily investigating miR-371 (Table 1). These trials comprise the majority of the current clinical trials investigating miRNA (Table 1). NCT05529251 is studying the use of miR-371 as a biomarker of response to determine its appropriateness for the de-escalation of treatment in stage IIa/IIb seminomas through correlation between miR-371 and fluorodeoxyglucose positron emission tomography results [95]. NCT06060873 and NCT04435756 are evaluating the accuracy of miR-371 in detecting active and relapsed disease in GCT [96,97]. NCT04914026 aims to assess the sensitivity and specificity of miR-371 in identifying viable GCT cells at the time of diagnosis and at the time of surgery and to evaluate miR-371 as a biomarker to monitor chemotherapy effectiveness and for early detection of recurrence [98]. Finally, NCT06133699 is evaluating the role of primary endoscopic sentinel lymph node biopsy in progression-free survival in patients with stage I-II GCTs without adjuvant treatment, along with assessing the miRNA expression profiles and prognostic values of miRNA as noninvasive markers in clinical practice [99].

## 6. Other Genitourinary Cancers

### 6.1. Penile Cancers

There were 36,068 new cases of penile cancers worldwide in 2020, and penile cancers may represent up to 10% of cancers in males in developing countries [100,101]. Approximately 95% of these are classified as penile squamous cell carcinoma (PSCC) [102]. PSCC is characterized by the uncontrolled proliferation of epithelial squamous cells and is often associated with human papillomavirus (HPV) [102]. Treatment options include organ-sparing approaches like topical therapy, wide local excision, laser therapy, and Mohs surgery for early-stage lesions [103]. Locally advanced stages may require perioperative chemotherapy, with local control consisting of either partial or total penectomy, radiotherapy, or even chemoradiotherapy, while for metastatic disease, systemic treatment is the only option. Those treatments include chemotherapy in the first-line setting, immunotherapy in subsequent lines, and epidermal growth factor receptor (EGFR) inhibitors in tumors with EGFR mutations [103]. Lymph node involvement is a key prognostic factor in PSCC; a pooled analysis of 217 patients with PSCC in two or fewer lymph nodes showed a 5-year survival rate of 77% compared with 25% in patients with greater nodal involvement [104].

Compared with other GU malignancies, there is little information about the use of miRNAs as effective biomarkers in penile cancer. Thus far, limited preclinical data highlight the potential of miRNA as a diagnostic and prognostic biomarker in penile cancer, but further studies are needed to validate these findings. Zhang et al. identified 56 differentially present miRNAs in penile cancer versus non-cancer tissues via next-generation sequencing [105]. Shortly after, Hartz et al. found that lower levels of miR-1, miR-101, and miR-204 can discriminate between patients with metastatic and localized PSCC [106]. Kuasne et al. performed an integrated analysis of mRNA and miRNA from samples in patients with PSCC and non-neoplastic tissues and found miR-31-5p, miR-223-3p, and miR-224-5p to be favorable biomarkers, as their overexpression was associated with metastasis [107]. A similar analysis performed by Marchi et al. identified that miR-31, miR-34a, and miR-130b overexpression is associated with metastasis, suggesting that these are promising biomarkers [108]. Furthermore, the upregulation of miR-223-3p and miR-107 in PSCC is associated with lymph node metastases and poorer prognosis, respectively [109]. Additionally, Murta et al. found that miR-744-5p and miR-421 were overexpressed in tissue samples of patients with metastatic PSCC, and a high expression of miR-421 was associated with lower overall survival [110].

Other studies have shown that miRNAs may be helpful in identifying HPV-dependent tumors. For instance, Ayoubian et al. performed a microarray between HPV-positive and HPV-negative PSCC samples and found 876 differentially present miRNAs [111]. From these, miR-99a-5p, miR-181d-5p, and miR-211-5p discriminated between tumor versus control tissue, and miR-137 and miR-328-3p differentiated metastatic from non-metastatic disease [111]. Additionally, Barzon et al. found that levels of miR-218 were higher in PSCC cases with high-risk HPV infection versus cases without high-risk HPV infection [112]. As research progresses, the identification of miRNAs as potential biomarkers highlights their relevance in clinical practice.

Evidence suggests that miRNAs could serve not only as diagnostic and prognostic tools, but also as novel therapeutic targets. For example, Furuya et al. revealed disruptions in the dynamics of mRNA–miRNA pairs during the development of PSCC [113]. These pairs represent key points in the regulatory network that have undergone alterations during tumor development [113]. Identifying specific dysregulated miRNA–mRNA pairs associated with penile carcinogenesis highlights potential targets for therapeutic intervention. MiR-145-5p expression is downregulated, correlating with the upregulation of its target mRNAs, including MMP1 and MCM2, in patients with lymph node involvement. MMPI and MCM2 are known contributors to cancer progression [113]. Targeting these dysregulated pairs could help restore normal regulatory mechanisms and impede tumor growth and progression. Similarly, Silva et al. studied the genomic landscape of HPV-positive penile cancer by analyzing copy number alterations and miRNA–mRNA interactions [114]. The enrichment pathways of mapped miRNAs revealed their involvement in the EGFR and cyclooxygenase-2 (COX-2) signaling pathways, both of which are implicated in tumor progression. The study indicates EGFR and COX2 as potential therapeutic targets in HPV-positive PSCC [114]. The exploration of miRNA-based therapeutic avenues and the identification of key molecular targets among these miRNAs offer promising potential for developing personalized and more effective treatment strategies for patients with PSCC.

### 6.2. Adrenocortical Tumors

Adrenocortical tumors (ACT) arise from the cortex of the adrenal gland and encompass benign adrenocortical adenomas (ACA) and malignant adrenocortical carcinomas (ACC). Globally, the estimated incidence of ACC approximates one case per million individuals annually; however, data on its epidemiology remain limited. Although rare, ACC is characterized by its poor prognosis, with 20–50% of patients presenting with advanced or metastatic disease, and overall survival is typically less than one year [115]. The treatment of early-stage ACC comprises open adrenalectomy, followed by administration of adjuvant mitotane; advanced stages may require aggressive surgical approaches [116]. Systemic therapy includes mitotane, either alone or in combination with chemotherapy such as carboplatin or cisplatin with etoposide [116]. For metastatic or refractory ACC, options include targeted therapies and immunotherapy for tumors with microsatellite instability or with high mutational burden [116].

Diagnosis of ACC is currently aided by hormonal, molecular, and genetic markers. However, these biomarkers often lack specificity and sensitivity, suffer from inter-individual variability, and can be invasive to measure. Additionally, their prognostic value is limited. miRNAs hold promise as biomarkers because of their tissue-specific expression and susceptibility to dysregulation in ACC. MiRNAs play roles in the initiation, proliferation, and progression of ACC and its targets. A study by Tömböl et al., the first to identify the miRNA expression profile of ACC, identified the upregulation of miR-503, miR-210, and miR-184, and the downregulation of miR-511, miR-214, and miR-375 in ACC samples compared to the levels in ACA samples [117]. MiR-503 exhibited the highest overexpression, and miR-511 displayed the most significant downregulation [117]. Similarly, Soon et al. studied a larger cohort and found reduced levels of miR-195 and miR-335 in ACC compared to ACA, with significant downregulation of miR-7 in both ACA and ACC compared to levels in the healthy adrenal tissue [118]. The distinct expression patterns of these miRNAs suggest their potential use as diagnostic tools to differentiate benign from malignant adrenal tumors.

Additionally, miRNAs have been evaluated as prognostic tools. Chabre et al. found that low levels of miR-195 were highly predictive and prognostic of aggressive ACC [119]. In another study, Patterson et al. found that patients with lower postoperative miR-483-5p levels after ACC resection showed significantly longer recurrence-free and overall survival rates compared to those with higher miR-483-5p levels [120]. Neither study identified the targets of these miRNAs [119,120].

Preclinical studies have identified potential miRNA-based therapeutic avenues in ACC. MiRNAs contribute to chemoresistance in ACC. Kwok et al. found downregulation of miR-341 in patients with metastatic ACC resistant to traditional therapies like doxorubicin, mitotane, and radiotherapy [121]. The authors identified the target of miR-431 as zinc finger E-box binding homeobox 1, and miR-431 reverses EMT when doxorubicin is administered [121]. Restoring miR-431 function in vitro sensitized ACC cells to these treatments, suggesting that miR-431 exhibits significant potential as a therapeutic agent to enhance the efficacy of chemotherapy in patients with resistant disease [121]. Bortoletto and Parchem explored the interplay between oncogenic Kirsten rat sarcoma viral oncogene homolog (KRAS) mutations and miRNA regulatory pathways in ACC [122]. KRAS mutations can hijack the miRNA biogenesis and processing machinery, leading to widespread miRNA misregulation that promotes ACC progression [122]. The researchers utilized RNA sequencing data from ACC samples to analyze miRNA expression patterns and examined how KRAS modulates key components of the miRNA machinery, such as RNase II/III Dicer enzymes and AGO2 [122]. Through biochemical assays and cellular models, they demonstrated that concurrent targeting of mutant KRAS and miRNA regulatory components could synergistically inhibit ACC cell growth and enhance chemosensitivity [122]. This approach suggests a novel and promising strategy for treating KRAS-driven ACCs.

A review by Igaz et al. describes both direct and indirect targeting of miRNAs in ACC [123]. Direct targeting involves using miRNA mimics to restore the function of downregulated tumor-suppressor miRNAs or employing antagomirs to inhibit upregulated oncogenic miRNAs [123]. Specifically, in ACC, miR-195 and miR-497 are underexpressed and act as tumor suppressors by targeting genes involved in cell cycle regulation and angiogenesis [123]. Indirect targeting includes modulating downstream pathways affected by miRNAs, such as the mTOR/Raptor and Notch signaling pathways, which are influenced by miRNAs like miR-99a and miR-100 in childhood ACC [123]. Igaz et al. utilized high-throughput screening and bioinformatics analysis to identify key miRNAs and their target pathways [123]. Experimental models, including ACC cell lines and animal studies, were used to validate the therapeutic potential of these approaches [123]. Despite the challenges of tissue-specific delivery and potential off-target effects, the findings underscore the promise of miRNA-based therapies in the treatment of ACC.

Two ongoing trials are exploring miRNA biomarkers and genetic factors in ACC (Table 1). NCT05660889 is investigating the use of adrenal vein sampling to identify biomarkers in ACC [124]. This study aims to improve diagnostic accuracy by analyzing blood samples for potential miRNAs and other biomarkers to better understand the molecular mechanisms underlying ACC [124]. NCT01528956 evaluated the genetic and molecular basis of these adrenal tumors through DNA methylation analysis, RNA analysis, and gene expression profiling to identify key genetic changes and potential therapeutic targets, including the involvement of miRNAs [125]. Trial enrollment has been completed, but the results are still pending. These studies highlight efforts to understand the role of miRNAs in ACC, and hopefully, additional studies investigating miRNAs as therapeutic agents are on the horizon.

## 7. Conclusions

MiRNAs regulate post-transcription gene expression through several mechanisms and are heavily implicated in the carcinogenesis of GU malignancies. Many preclinical studies have demonstrated the utility of miRNAs as biomarkers or diagnostic tools, and in vitro and in vivo studies have evaluated synthetic miRNAs to promote tumor suppression or inhibit oncogenesis. Clinical studies are ongoing, mostly to evaluate miRNAs as biomarkers, although some trials using miRNAs as therapeutic agents are in process. It is evident that not all cancers are equal, and as we move toward an era of personalized medicine, we strive to create individualized cancer treatment plans that best suit each patient. MiRNAs can play a crucial role in predicting which patients will respond to specific therapeutic agents, with the potential to truly revolutionize cancer care. With several studies in the pipeline, miRNAs are an exciting new frontier under investigation in GU malignancies, and miRNAs may be the preferred diagnostic, prognostic, and therapeutic agents in the coming years.

## Figures and Tables

**Table 1:** Ongoing clinical trials involving miRNAs in GU malignancies at the time of publication.

Clinical Trial Number	Study Type	miRNA	Malignancy	Brief Description
NCT05529251	Interventional, phase II	miR-371a-3p	Seminoma, stage IIa/IIb	Investigating the correlation between miR-371 as a biomarker using positron emission tomography (PET) scanning as a tool for de-escalating treatment.
NCT06060873	Observational	miR-371	Active germ cell tumor	Investigating miR-371 as a biomarker for optimal timing of retroperitoneal lymph node dissection (RPLND).
NCT04435756	Observational	miR-371	Early-stage testicular seminoma and nonseminoma	Investigating the positive predictive value of miR-371 as a marker of disease relapse.
NCT04914026	Observational	miR-371a-5p	All testicular germ cell tumors	Investigating the sensitivity and specificity of miR-371 as a biomarker at orchiectomy and during treatment and surveillance.
NCT06133699	Interventional	MiRNA expression profiles	Germ cell tumors, stage IA/IIA	Investigating miRNA expression profiles as a prognostic tool throughout treatment.
NCT05660889	Observational	MiRNA expression profiles	Adrenocortical carcinoma	Investigating miRNAs as diagnostic biomarkers.
NCT01528956	Observational	MiRNA expression profiles	Pediatric adrenocortical tumors	Investigating miRNA expression in tumor cells compared to normal cells.

## Data Availability

Not applicable.

## References

[1] Lee L.W., Zhang S., Etheridge A., Ma L., Martin D., Galas D., Wang K. (2010). Complexity of the microRNA repertoire revealed by next-generation sequencing. RNA.

[2] Lytle J.R., Yario T.A., Steitz J.A. (2007). Target mRNAs are repressed as efficiently by microRNA-binding sites in the 5′ UTR as in the 3′ UTR. Proc. Natl. Acad. Sci. USA.

[3] Alles J., Fehlmann T., Fischer U., Backes C., Galata V., Minet M., Hart M., Abu-Halima M., Grässer F.A., Lenhof H.-P. (2019). An estimate of the total number of true human miRNAs. Nucleic Acids Res..

[4] Lee R.C., Feinbaum R.L., Ambros V. (1993). The *C. elegans* heterochronic gene *lin-4* encodes small RNAs with antisense complementarity to *lin-14*. Cell.

[5] Vasudevan S., Tong Y., Steitz J.A. (2007). Switching from Repression to Activation: MicroRNAs Can Up-Regulate Translation. Science.

[6] Lotterman C.D., Kent O.A., Mendell J.T. (2008). Functional integration of microRNAs into oncogenic and tumor suppressor pathways. Cell Cycle.

[7] Tay F.C., Lim J.K., Zhu H., Hin L.C., Wang S. (2015). Using artificial microRNA sponges to achieve microRNA loss-of-function in cancer cells. Adv. Drug Deliv. Rev..

[8] Beltrán-García J., Osca-Verdegal R., Mena-Mollá S., García-Giménez J.L. (2019). Epigenetic IVD Tests for Personalized Precision Medicine in Cancer. Front. Genet..

[9] Calin G.A., Sevignani C., Dumitru C.D., Hyslop T., Noch E., Yendamuri S., Shimizu M., Rattan S., Bullrich F., Negrini M. (2004). Human microRNA genes are frequently located at fragile sites and genomic regions involved in cancers. Proc. Natl. Acad. Sci. USA.

[10] Tian Y.-Q., Yang J.-C., Hu J.-J., Ding R., Ye D.-W., Shang J.-W. (2023). Trends and risk factors of global incidence, mortality, and disability of genitourinary cancers from 1990 to 2019: Systematic analysis for the Global Burden of Disease Study 2019. Front. Public Health.

[11] Catto J.W.F., Miah S., Owen H.C., Bryant H., Myers K., Dudziec E., Larré S., Milo M., Rehman I., Rosario D.J. (2009). Distinct MicroRNA Alterations Characterize High- and Low-Grade Bladder Cancer. Cancer Res..

[12] Antolín S., Calvo L., Blanco-Calvo M., Santiago M.P., Lorenzo-Patiño M.J., Haz-Conde M., Santamarina I., Figueroa A., Antón-Aparicio L.M., Valladares-Ayerbes M. (2015). Circulating miR-200c and miR-141 and outcomes in patients with breast cancer. BMC Cancer.

[13] National Cancer Institute Surveillance, Epidemiology, and End Results Program (SEER). Cancer Stat Facts: Prostate Cancer. https://seer.cancer.gov/statfacts/html/prost.html.

[14] Humphrey P.A., Moch H., Cubilla A.L., Ulbright T.M., Reuter V.E. (2016). The 2016 WHO Classification of Tumours of the Urinary System and Male Genital Organs—Part B: Prostate and Bladder Tumours. Eur. Urol..

[15] National Comprehensive Cancer Network (2020). “Prostate Cancer”. https://www.nccn.org/professionals/physician_gls/pdf/prostate.pdf.

[16] Sartor O., de Bono J., Chi K.N., Fizazi K., Herrmann K., Rahbar K., Tagawa S.T., Nordquist L.T., Vaishampayan N., El-Haddad G. (2021). Lutetium-177–PSMA-617 for Metastatic Castration-Resistant Prostate Cancer. N. Engl. J. Med..

[17] Filella X., Fernández-Galán E., Bonifacio R.F., Foj L. (2018). Emerging biomarkers in the diagnosis of prostate cancer. Pharmacogenom. Pers. Med..

[18] Kanwal R., Plaga A.R., Liu X., Shukla G.C., Gupta S. (2017). MicroRNAs in prostate cancer: Functional role as biomarkers. Cancer Lett..

[19] Zhang B., Pan X., Cobb G., Anderson T. (2007). microRNAs As oncogenes and tumor suppressors. Dev. Biol..

[20] Haldrup C., Kosaka N., Ochiya T., Borre M., Høyer S., Orntoft T.F., Sorensen K.D. (2013). Profiling of circulating microRNAs for prostate cancer biomarker discovery. Drug Deliv. Transl. Res..

[21] Brase J.C., Johannes M., Schlomm T., Fälth M., Haese A., Steuber T., Beissbarth T., Kuner R., Sültmann H. (2010). Circulating miRNAs are correlated with tumor progression in prostate cancer. Int. J. Cancer.

[22] Cheng H.H., Plets M., Li H., Higano C.S., Tangen C.M., Agarwal N., Vogelzang N.J., Hussain M., Thompson I.M., Tewari M. (2018). Circulating microRNAs and treatment response in the Phase II SWOG S0925 study for patients with new metastatic hormone-sensitive prostate cancer. Prostate.

[23] Walter B.A., Valera V.A., Pinto P.A., Merino M.J. (2013). Comprehensive microRNA Profiling of Prostate Cancer. J. Cancer.

[24] Mishra S., Deng J.J., Gowda P.S., Rao M.K., Lin C.-L., Chen C.L., Huang T., Sun L.-Z. (2013). Androgen receptor and microRNA-21 axis downregulates transforming growth factor beta receptor II (TGFBR2) expression in prostate cancer. Oncogene.

[25] E Jalava S., Urbanucci A., Latonen L., Waltering K.K., Sahu B., A Jänne O., Seppälä J., Lähdesmäki H., Tammela T.L.J., Visakorpi T. (2012). Androgen-regulated miR-32 targets BTG2 and is overexpressed in castration-resistant prostate cancer. Oncogene.

[26] Wan X., Pu H., Huang W., Yang S., Zhang Y., Kong Z., Yang Z., Zhao P., Li A., Li T. (2016). Androgen-induced miR-135a acts as a tumor suppressor through downregulating RBAK and MMP11, and mediates resistance to androgen deprivation therapy. Oncotarget.

[27] Li F., Mahato R.I. (2014). MicroRNAs and Drug Resistance in Prostate Cancers. Mol. Pharm..

[28] Lima J.F., Cerqueira L., Figueiredo C., Oliveira C., Azevedo N.F. (2018). Anti-miRNA oligonucleotides: A comprehensive guide for design. RNA Biol..

[29] Krützfeldt J., Rajewsky N., Braich R., Rajeev K.G., Tuschl T., Manoharan M., Stoffel M. (2005). Silencing of microRNAs in vivo with ‘antagomirs’. Nature.

[30] Mercatelli N., Coppola V., Bonci D., Miele F., Costantini A., Guadagnoli M., Bonanno E., Muto G., Frajese G.V., De Maria R. (2008). The Inhibition of the Highly Expressed Mir-221 and Mir-222 Impairs the Growth of Prostate Carcinoma Xenografts in Mice. PLoS ONE.

[31] Mollaei H., Safaralizadeh R., Rostami Z. (2019). MicroRNA replacement therapy in cancer. J. Cell. Physiol..

[32] Gaur S., Wen Y., Song J.H., Parikh N.U., Mangala L.S., Blessing A.M., Ivan C., Wu S.Y., Varkaris A., Shi Y. (2015). Chitosan nanoparticle-mediated delivery of miRNA-34a decreases prostate tumor growth in the bone and its expression induces non-canonical autophagy. Oncotarget.

[33] Hao Z., Fan W., Hao J., Wu X., Zeng G.Q., Zhang L.J., Nie S.F., Wang X.D. (2014). Efficient delivery of micro RNA to bone-metastatic prostate tumors by using aptamer-conjugated atelocollagen in vitro and in vivo. Drug Deliv..

[34] Bennett C.F., Baker B.F., Pham N., Swayze E., Geary R.S. (2017). Pharmacology of Antisense Drugs. Annu. Rev. Pharmacol. Toxicol..

[35] van Hoogstraten L.M.C., Vrieling A., van der Heijden A.G., Kogevinas M., Richters A., Kiemeney L.A. (2023). Global trends in the epidemiology of bladder cancer: Challenges for public health and clinical practice. Nat. Rev. Clin. Oncol..

[36] Meng W., Efstathiou J., Singh R., McElroy J., Volinia S., Cui R., Ibrahim A., Johnson B., Gupta N., Mehta S. (2018). MicroRNA Biomarkers for Patients with Muscle-Invasive Bladder Cancer Undergoing Selective Bladder-Sparing Trimodality Treatment. Int. J. Radiat. Oncol..

[37] Amir S., Mabjeesh N.J. (2017). microRNA Expression profiles as decision-making biomarkers in the management of bladder cancer. Histol. Histopathol..

[38] Browne B.M., Stensland K.D., Patel C.K., Sullivan T., Burks E.J., Canes D., Raman J.D., Warrick J., Reiger-Christ K.M. (2019). MicroRNA Expression Profiles in Upper Tract Urothelial Carcinoma Differentiate Tumor Grade, Stage, and Survival: Implications for Clinical Decision-Making. Urology.

[39] Xie Y., Ma X., Chen L., Li H., Gu L., Gao Y., Zhang Y., Li X., Fan Y., Chen J. (2017). MicroRNAs with prognostic significance in bladder cancer: A systematic review and meta-analysis. Sci. Rep..

[40] Rosenberg E., Baniel J., Spector Y., Faerman A., Meiri E., Aharonov R., Margel D., Goren Y., Nativ O. (2013). Predicting progression of bladder urothelial carcinoma using microRNA expression. BJU Int..

[41] Aveta A., Cilio S., Contieri R., Spena G., Napolitano L., Manfredi C., Franco A., Crocerossa F., Cerrato C., Ferro M. (2023). Urinary MicroRNAs as Biomarkers of Urological Cancers: A Systematic Review. Int. J. Mol. Sci..

[42] Pilala K.-M., Kotronopoulos G., Levis P., Giagkos G.-C., Stravodimos K., Vassilacopoulou D., Scorilas A., Avgeris M. (2024). *MIR145* Core Promoter Methylation in Pretreatment Cell-Free DNA: A Liquid Biopsy Tool for Muscle-Invasive Bladder Cancer Treatment Outcome. JCO Precis. Oncol..

[43] Xu C., Zeng Q., Xu W., Jiao L., Chen Y., Zhang Z., Wu C., Jin T., Pan A., Wei R. (2013). miRNA-100 Inhibits Human Bladder Urothelial Carcinogenesis by Directly Targeting mTOR. Mol. Cancer Ther..

[44] Uchino K., Takeshita F., Takahashi R.-U., Kosaka N., Fujiwara K., Naruoka H., Sonoke S., Yano J., Sasaki H., Nozawa S. (2013). Therapeutic Effects of MicroRNA-582-5p and -3p on the Inhibition of Bladder Cancer Progression. Mol. Ther..

[45] Wang Y., Xing Q.-F., Liu X.-Q., Guo Z.-J., Li C.-Y., Sun G. (2016). MiR-122 targets VEGFC in bladder cancer to inhibit tumor growth and angiogenesis. Am. J. Transl. Res..

[46] Wang X., Wu Q., Xu B., Wang P., Fan W., Cai Y., Gu X., Meng F. (2015). miR-124 Exerts tumor suppressive functions on the cell proliferation, motility and angiogenesis of bladder cancer by fine-tuning UHRF1. FEBS J..

[47] Drakaki A., Polytarchou C., O’Brien N.A., Iliopoulos D., Slamon D.J. (2015). Role and therapeutic targeting of miR-21 in bladder cancer. J. Clin. Oncol..

[48] Drakaki A., Koutsioumpa M., O’Brien N.A., Vorvis C., Iliopoulos D., Slamon D.J. (2017). A chemically-modified miR-21 inhibitor (ADM-21) as a novel potential therapy in bladder cancer. J. Clin. Oncol..

[49] Koutsioumpa M., Chen H.-W., O’Brien N., Koinis F., Mahurkar-Joshi S., Vorvis C., Soroosh A., Luo T., Issakhanian S., Pantuck A.J. (2018). MKAD-21 Suppresses the Oncogenic Activity of the miR-21/PPP2R2A/ERK Molecular Network in Bladder Cancer. Mol. Cancer Ther..

[50] Heishima K., Sugito N., Abe C., Hirata A., Sakai H., Akao Y. (2023). Targeting microRNA-145-mediated progressive phenotypes of early bladder cancer in a molecularly defined in vivo model. Mol. Ther.-Nucleic Acids.

[51] Hong D.S., Kang Y.K., Borad M., Sachdev J., Ejadi S., Lim H.Y., Brenner A.J., Park K., Lee J.L., Kim T.Y. (2020). Phase 1 study of MRX34, a liposomal miR-34a mimic, in patients with advanced solid tumours. Br. J. Cancer.

[52] Padala S.A., Barsouk A., Thandra K.C., Saginala K., Mohammed A., Vakiti A., Rawla P., Barsouk A. (2020). Epidemiology of Renal Cell Carcinoma. World J. Oncol..

[53] NCCN Guidelines Detail. https://www.nccn.org/guidelines/guidelines-detail.

[54] Kathuria-Prakash N., Drolen C., Hannigan C.A., Drakaki A. (2021). Immunotherapy and Metastatic Renal Cell Carcinoma: A Review of New Treatment Approaches. Life.

[55] Elballal M.S., Sallam A.-A.M., Elesawy A.E., Shahin R.K., Midan H.M., Elrebehy M.A., Elazazy O., El-Boghdady R.M., Blasy S.H., Amer N.M. (2023). miRNAs As potential game-changers in renal cell carcinoma: Future clinical and medicinal uses. Pathol.-Res. Pract..

[56] Tusong H., Maolakuerban N., Guan J., Rexiati M., Wang W.-G., Azhati B., Nuerrula Y., Wang Y.-J. (2017). Functional analysis of serum microRNAs miR-21 and miR-106a in renal cell carcinoma. Cancer Biomark..

[57] Petillo D. (2009). MicroRNA profiling of human kidney cancer subtypes. Int. J. Oncol..

[58] Xiao W., Lou N., Ruan H., Bao L., Xiong Z., Yuan C., Tong J., Xu G., Zhou Y., Qu Y. (2017). Mir-144-3p Promotes Cell Proliferation, Metastasis, Sunitinib Resistance in Clear Cell Renal Cell Carcinoma by Downregulating ARID1A. Cell. Physiol. Biochem..

[59] Berkers J., Govaere O., Wolter P., Beuselinck B., Schöffski P., van Kempen L.C., Albersen M., Oord J.V.D., Roskams T., Swinnen J. (2012). A Possible Role for MicroRNA-141 Down-Regulation in Sunitinib Resistant Metastatic Clear Cell Renal Cell Carcinoma through Induction of Epithelial-to-Mesenchymal Transition and Hypoxia Resistance. J. Urol..

[60] Sekino Y., Sakamoto N., Sentani K., Oue N., Teishima J., Matsubara A., Yasui W. (2019). miR-130b Promotes Sunitinib Resistance through Regulation of PTEN in Renal Cell Carcinoma. Oncology.

[61] Park S.E., Kim W., Hong J.-Y., Kang D., Park S., Suh J., You D., Park Y.-Y., Suh N., Hwang J.J. (2022). miR-96-5p Targets PTEN to mediate sunitinib resistance in clear cell renal cell carcinoma. Sci. Rep..

[62] He J., He J., Min L., He Y., Guan H., Wang J., Peng X. (2019). Extracellular vesicles transmitted miR-31-5p promotes sorafenib resistance by targeting MLH1 in renal cell carcinoma. Int. J. Cancer.

[63] Gao C., Peng F.H., Peng L.K. (2014). MiR-200c sensitizes clear-cell renal cell carcinoma cells to sorafenib and imatinib by targeting heme oxygenase-1. Neoplasma.

[64] Chen S., Wang L., Yao X., Chen H., Xu C., Tong L., Shah A., Huang T., Chen G., Chen J. (2017). miR-195-5p Is critical in REGγ-mediated regulation of wnt/β-catenin pathway in renal cell carcinoma. Oncotarget.

[65] Yang H., Song E., Shen G., Zhu T., Jiang T., Shen H., Niu L., Wang B., Lu Z., Qian J. (2017). Expression of microRNA-30c via lentivirus vector inhibits the proliferation and enhances the sensitivity of highly aggressive ccRCC Caki-1 cells to anticancer agents. OncoTargets Ther..

[66] Sun X., Lou L., Zhong K., Wan L. (2017). MicroRNA-451 regulates chemoresistance in renal cell carcinoma by targeting ATF-2 gene. Exp. Biol. Med..

[67] Chen L., Chen L., Qin Z., Lei J., Ye S., Zeng K., Wang H., Ying M., Gao J., Zeng S. (2019). Upregulation of miR-489-3p and miR-630 inhibits oxaliplatin uptake in renal cell carcinoma by targeting OCT2. Acta Pharm. Sin. B.

[68] Gaudelot K., Gibier J.-B., Pottier N., Hémon B., Van Seuningen I., Glowacki F., Leroy X., Cauffiez C., Gnemmi V., Aubert S. (2017). Targeting miR-21 decreases expression of multi-drug resistant genes and promotes chemosensitivity of renal carcinoma. Tumor Biol..

[69] Chen B., Duan L., Yin G., Tan J., Jiang X. (2013). miR-381, A novel intrinsic WEE1 inhibitor, sensitizes renal cancer cells to 5-FU by up-regulation of Cdc2 activities in 786-O. J. Chemother..

[70] Ivanova E., Asadullina D., Gilyazova G., Rakhimov R., Izmailov A., Pavlov V., Khusnutdinova E., Gilyazova I. (2023). Exosomal MicroRNA Levels Associated with Immune Checkpoint Inhibitor Therapy in Clear Cell Renal Cell Carcinoma. Biomedicines.

[71] Zhao J., Lei T., Xu C., Li H., Ma W., Yang Y., Fan S., Liu Y. (2013). MicroRNA-187, down-regulated in clear cell renal cell carcinoma and associated with lower survival, inhibits cell growth and migration though targeting B7-H3. Biochem. Biophys. Res. Commun..

[72] Kalantzakos T., Hooper K., Das S., Sullivan T., Canes D., Moinzadeh A., Rieger-Christ K. (2023). MicroRNA-155-5p Targets JADE-1, Promoting Proliferation, Migration, and Invasion in Clear Cell Renal Cell Carcinoma Cells. Int. J. Mol. Sci..

[73] Liang T., Hu X.-Y., Li Y.-H., Tian B.-Q., Li Z.-W., Fu Q. (2016). MicroRNA-21 Regulates the Proliferation, Differentiation, and Apoptosis of Human Renal Cell Carcinoma Cells by the mTOR-STAT3 Signaling Pathway. Oncol. Res. Featur. Preclin. Clin. Cancer Ther..

[74] Zhang Q., Ren H., Ge L., Zhang W., Song F., Huang P. (2023). A review on the role of long non-coding RNA and microRNA network in clear cell renal cell carcinoma and its tumor microenvironment. Cancer Cell Int..

[75] Schiffgen M., Schmidt D.H., von Rücker A., Müller S.C., Ellinger J. (2013). Epigenetic regulation of microRNA expression in renal cell carcinoma. Biochem. Biophys. Res. Commun..

[76] Wu A., Wu K., Li M., Bao L., Shen X., Li S., Li J., Yang Z. (2012). Upregulation of microRNA-492 induced by epigenetic drug treatment inhibits the malignant phenotype of clear cell renal cell carcinoma in vitro. Mol. Med. Rep..

[77] Gaddam S., Chesnut G. (2023). Testicular Cancer.

[78] Smith Z.L., Werntz R.P., Eggener S.E. (2018). Testicular Cancer. Med. Clin. N. Am..

[79] Global Cancer Observatory Testis Fact Sheet. International Agency for Research on Cancer, World Health Organization..

[80] Goodall G.J., Wickramasinghe V.O. (2020). RNA in cancer. Nat. Rev. Cancer.

[81] Nestler T., Schoch J., Belge G., Dieckmann K.-P. (2023). MicroRNA-371a-3p—The Novel Serum Biomarker in Testicular Germ Cell Tumors. Cancers.

[82] Murray M.J., Halsall D.J., Hook C.E., Williams D.M., Nicholson J.C., Coleman N. (2011). Identification of MicroRNAs From the miR-371∼373 and miR-302 Clusters as Potential Serum Biomarkers of Malignant Germ Cell Tumors. Am. J. Clin. Pathol..

[83] van Agthoven T., Looijenga L.H. (2016). Accurate primary germ cell cancer diagnosis using serum based microRNA detection (ampTSmiR test). Oncotarget.

[84] Dieckmann K.-P., Radtke A., Spiekermann M., Balks T., Matthies C., Becker P., Ruf C., Oing C., Oechsle K., Bokemeyer C. (2016). Serum Levels of MicroRNA miR-371a-3p: A Sensitive and Specific New Biomarker for Germ Cell Tumours. Eur. Urol..

[85] Lobo J., Gillis A.J.M., van den Berg A., Dorssers L.C.J., Belge G., Dieckmann K.-P., Roest H.P., Van Der Laan L.J.W., Gietema J., Hamilton R.J. (2019). Identification and Validation Model for Informative Liquid Biopsy-Based microRNA Biomarkers: Insights from Germ Cell Tumor In Vitro, In Vivo and Patient-Derived Data. Cells.

[86] Belge G., Dumlupinar C., Nestler T., Klemke M., Törzsök P., Trenti E., Pichler R., Loidl W., Che Y., Hiester A. (2023). Detection of Recurrence through microRNA-371a-3p Serum Levels in a Follow-up of Stage I Testicular Germ Cell Tumors in the DRKS-00019223 Study. Clin. Cancer Res..

[87] Li H.-L., Wei J.-F., Fan L.-Y., Wang S.-H., Zhu L., Li T.-P., Lin G., Sun Y., Sun Z.-J., Ding J. (2016). miR-302 Regulates pluripotency, teratoma formation and differentiation in stem cells via an AKT1/OCT4-dependent manner. Cell Death Dis..

[88] Gao Z., Zhu X., Dou Y. (2015). The miR-302/367 cluster: A comprehensive update on its evolution and functions. Open Biol..

[89] Das M.K., Evensen H.S.F., Furu K., Haugen T.B. (2019). miRNA-302s May act as oncogenes in human testicular germ cell tumours. Sci. Rep..

[90] Syring I., Bartels J., Holdenrieder S., Kristiansen G., Müller S.C., Ellinger J. (2015). Circulating Serum miRNA (miR-367-3p, miR-371a-3p, miR-372-3p and miR-373-3p) as Biomarkers in Patients with Testicular Germ Cell Cancer. J. Urol..

[91] Lin Y.-H., Su C.-H., Chen H.-M., Wu M.-S., Pan H.-A., Chang C.-N., Cheng Y.-S., Chang W.-T., Chiu C.-C., Teng Y.-N. (2024). MicroRNA-320a enhances LRWD1 expression through the AGO2/FXR1-dependent pathway to affect cell behaviors and the oxidative stress response in human testicular embryonic carcinoma cells. Aging.

[92] Li H., Yu L., Liu J., Bian X., Shi C., Sun C., Zhou X., Wen Y., Hua D., Zhao S. (2017). miR-320a Functions as a suppressor for gliomas by targeting SND1 and β-catenin, and predicts the prognosis of patients. Oncotarget.

[93] Sun J.-Y., Zhao Z.-W., Li W.-M., Yang G., Jing P.-Y., Li P., Dang H.-Z., Chen Z., Zhou Y.-A., Li X.-F. (2017). Knockdown of MALAT1 expression inhibits HUVEC proliferation by upregulation of miR-320a and downregulation of FOXM1 expression. Oncotarget.

[94] Elesawy A.E., Abulsoud A.I., Moustafa H.A.M., Elballal M.S., Sallam A.-A.M., Elazazy O., El-Dakroury W.A., Mageed S.S.A., Abdelmaksoud N.M., Midan H.M. (2023). miRNAs Orchestration of testicular germ cell tumors—Particular emphasis on diagnosis, progression and drug resistance. Pathol.-Res. Pract..

[95] National Library of Medicine Prospective Therapeutic De-Escalation and miRNA-M371 Biomarker Evaluation Phase II Study for Stage IIa/IIb <3 cm Seminomas. ClinicalTrials.gov identifier: NCT05529251. 25 January 2024. NCT05529251.

[96] National Library of Medicine MAGESTIC Trial: MiRNA in Detecting Active Germ Cell Tumors in Early Suspected and Meta-staTIC Disease Trial. ClinicalTrials.gov identifier: NCT06060873. 3 November 2023. NCT06060873.

[97] National Library of Medicine A Prospective Observational Cohort Study to Assess miRNA 371 for Outcome Prediction in Pa-tients with Newly Diagnosed Germ Cell Tumors. ClinicalTrials.gov identifier: NCT04435756. 12 December 2023. NCT04435756.

[98] National Library of Medicine MicroRNA-371 as Markers for Disease Activity and as a Tool to Monitor the Effect of Chemo-therapy and Early Detection of Recurrence in Patients with Testicular Germ Cell Tumours. ClinicalTrials.gov identifier: NCT04914026. 8 August 2022. NCT04914026.

[99] National Library of Medicine A Prospective, Single-Center, Clinical Trial to Evaluate the Efficacy of Sentinel Lymph Node Bi-opsy in Stage AI-IIA Germ Cell Tumors (Seminoma/Nonseminoma) (PITERLAND). ClinicalTrials.gov identifier: NCT06133699. 3 January 2024. NCT06133699.

[100] Sung H., Ferlay J., Siegel R.L., Laversanne M., Soerjomataram I., Jemal A., Bray F. (2021). Global Cancer Statistics 2020: GLOBOCAN Estimates of Incidence and Mortality Worldwide for 36 Cancers in 185 Countries. CA Cancer J. Clin..

[101] Pow-Sang M.R., Ferreira U., Pow-Sang J.M., Nardi A.C., Destefano V. (2010). Epidemiology and Natural History of Penile Cancer. Urology.

[102] Giona S., Barber N., Ali A. (2022). The Epidemiology of Penile Cancer. Urologic Cancers.

[103] National Comprehemsive Cancer Network Penile Cancer Guidelines. 25 October 2023. https://www.nccn.org/professionals/physician_gls/pdf/penile.pdf.

[104] Zhu Y., Ye D.-W. (2012). Lymph node metastases and prognosis in penile cancer. Chin. J. Cancer Res..

[105] Zhang L., Wei P., Shen X., Zhang Y., Xu B., Zhou J., Fan S., Hao Z., Shi H., Zhang X. (2015). MicroRNA Expression Profile in Penile Cancer Revealed by Next-Generation Small RNA Sequencing. PLoS ONE.

[106] Hartz J.M., Engelmann D., Fürst K., Marquardt S., Spitschak A., Goody D., Protzel C., Hakenberg O.W., Pützer B.M. (2016). Integrated Loss of miR-1/miR-101/miR-204 Discriminates Metastatic from Nonmetastatic Penile Carcinomas and Can Predict Patient Outcome. J. Urol..

[107] Kuasne H., Barros-Filho M.C., Busso-Lopes A., Marchi F.A., Pinheiro M., Muñoz J.J.M., Scapulatempo-Neto C., Faria E.F., Guimarães G.C., Lopes A. (2017). Integrative miRNA and mRNA analysis in penile carcinomas reveals markers and pathways with potential clinical impact. Oncotarget.

[108] Marchi F.A., Martins D.C., Barros-Filho M.C., Kuasne H., Lopes A.F.B., Brentani H., Filho J.C.S.T., Guimarães G.C., Faria E.F., Scapulatempo-Neto C. (2017). Multidimensional integrative analysis uncovers driver candidates and biomarkers in penile carcinoma. Sci. Rep..

[109] Pinho J.D., Silva G.E.B., Júnior A.A.L.T., Belfort M.R.d.C., Mendes J.M., da Cunha I.W., Quintana L.G., Calixto J.d.R.R., Nogueira L.R., Coelho R.W.P. (2020). MIR-107, MIR-223-3P and MIR-21-5P Reveals Potential Biomarkers in Penile Cancer. Asian Pac. J. Cancer Prev..

[110] Murta C.B., Furuya T.K., Carrasco A.G.M., Uno M., Sichero L., Villa L.L., Faraj S.F., Coelho R.F., Guglielmetti G.B., Cordeiro M.D. (2022). miRNA And mRNA Expression Profiles Associated with Lymph Node Metastasis and Prognosis in Penile Carcinoma. Int. J. Mol. Sci..

[111] Ayoubian H., Heinzelmann J., Hölters S., Khalmurzaev O., Pryalukhin A., Loertzer P., Heinzelbecker J., Lohse S., Geppert C., Loertzer H. (2021). miRNA Expression Characterizes Histological Subtypes and Metastasis in Penile Squamous Cell Carcinoma. Cancers.

[112] Barzon L., Cappellesso R., Peta E., Militello V., Sinigaglia A., Fassan M., Simonato F., Guzzardo V., Ventura L., Blandamura S. (2014). Profiling of Expression of Human Papillomavirus–Related Cancer miRNAs in Penile Squamous Cell Carcinomas. Am. J. Pathol..

[113] Furuya T.K., Murta C.B., Carrasco A.G.M., Uno M., Sichero L., Villa L.L., Cardilli L., Coelho R.F., Guglielmetti G.B., Cordeiro M.D. (2021). Disruption of miRNA-mRNA Networks Defines Novel Molecular Signatures for Penile Carcinogenesis. Cancers.

[114] da Silva J., Nogueira L., Coelho R., Deus A., Khayat A., Marchi R., de Oliveira E., dos Santos A.P., Cavalli L., Pereira S. (2021). HPV-associated penile cancer: Impact of copy number alterations in miRNA/mRNA interactions and potential druggable targets. Cancer Biomark..

[115] Allolio B., Fassnacht M. (2006). Adrenocortical Carcinoma: Clinical Update. J. Clin. Endocrinol. Metab..

[116] National Comprehensive Cancer Network Neuroendocrine and Adrenal Tumors Guidelines. 20 June 2024. https://www.nccn.org/professionals/physician_gls/pdf/neuroendocrine.pdf.

[117] Tömböl Z., Szabó P.M., Molnár V., Wiener Z., Tölgyesi G., Horányi J., Riesz P., Reismann P., Patócs A., Likó I. (2009). Integrative molecular bioinformatics study of human adrenocortical tumors: microRNA, tissue-specific target prediction, and pathway analysis. Endocr.-Relat. Cancer.

[118] Soon P.S.H., Tacon L.J., Gill A.J., Bambach C.P., Sywak M.S., Campbell P.R., Yeh M.W., Wong S.G., Clifton-Bligh R.J., Robinson B.G. (2009). miR-195 And miR-483-5p Identified as Predictors of Poor Prognosis in Adrenocortical Cancer. Clin. Cancer Res..

[119] Chabre O., Libé R., Assie G., Barreau O., Bertherat J., Bertagna X., Feige J.-J., Cherradi N. (2013). Serum miR-483-5p and miR-195 are predictive of recurrence risk in adrenocortical cancer patients. Endocr.-Relat. Cancer.

[120] Patterson E.E., Holloway A.K., Weng J., Fojo T., Kebebew E. (2010). MicroRNA profiling of adrenocortical tumors reveals miR-483 as a marker of malignancy. Cancer.

[121] Kwok G.T., Zhao J.T., Glover A.R., Gill A.J., Clifton-Bligh R., Robinson B.G., Ip J.C., Sidhu S.B. (2019). microRNA-431 As a Chemosensitizer and Potentiator of Drug Activity in Adrenocortical Carcinoma. Oncologist.

[122] Bortoletto A.S., Parchem R.J. (2023). KRAS Hijacks the miRNA Regulatory Pathway in Cancer. Cancer Res..

[123] Igaz P., Igaz I., Nagy Z., Nyírő G., Szabó P.M., Falus A., Patócs A., Rácz K. (2014). MicroRNAs in adrenal tumors: Relevance for pathogenesis, diagnosis, and therapy. Cell. Mol. Life Sci..

[124] National Library of Medicine Adrenal Vein Sampling as a Tool to Identify Biomarkers That Aid the Diagnosis of Adrenocortical Carcinoma (AVS for ACC) NCT05660889. 21 December 2022. NCT05660889.

[125] National Library of Medicine Studying Genes in Samples From Younger Patients with Adrenocortical Tumor NCT01528956. 18 May 2016. NCT01528956.

